# One-Pot Time-Induced Proteome Integral Solubility Alteration Assay for Automated and Sensitive Drug–Target Identification

**DOI:** 10.1021/acs.analchem.4c05127

**Published:** 2024-11-20

**Authors:** Zhaowei Meng, Amir Ata Saei, Hezheng Lyu, Massimiliano Gaetani, Roman A. Zubarev

**Affiliations:** † Division of Chemistry I, Department of Medical Biochemistry and Biophysics, Karolinska Institutet, 17177 Stockholm, Sweden; ‡ Chemical Proteomics Unit, Science for Life Laboratory (SciLifeLab), 17165 Stockholm, Sweden; § Chemical Proteomics, Swedish National Infrastructure for Biological Mass Spectrometry (BioMS), 17177 Stockholm, Sweden; ∥ Department of Microbiology, Tumor and Cell Biology, Karolinska Institutet, 17177 Stockholm, Sweden; ⊥ Biomotif AB, 18212 Danderyd, Sweden; # Department of Pharmaceutical and Toxicological Chemistry, Medical Institute, Peoples’ Friendship University of Russia named after Patrice Lumumba (RUDN University), 6 Miklukho-Maklaya St., Moscow 117198, Russian Federation

## Abstract

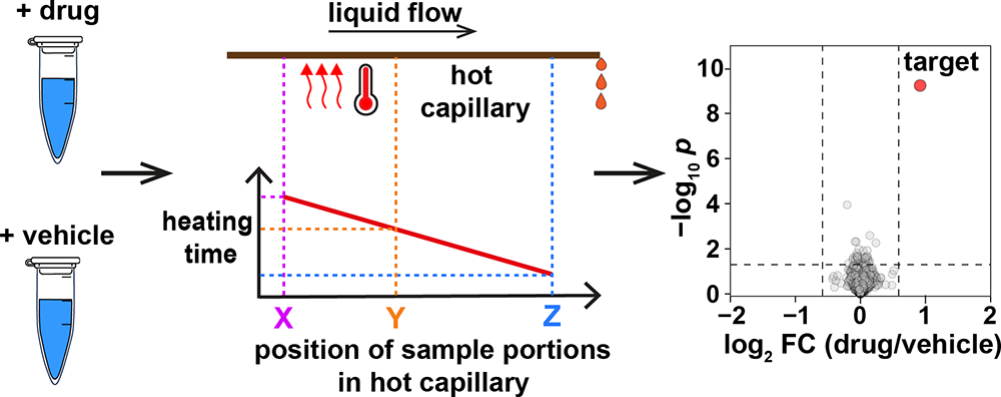

The proteome integral solubility alteration (PISA) assay is widely used for identifying drug targets, but it is labor-intensive and time-consuming and requires a substantial amount of biological sample. Aiming at enabling automation and greatly reducing the sample amount, we developed one-pot time-induced (OPTI)-PISA. Here, we demonstrate OPTI-PISA performance on identifying targets of multiple drugs in cell lysate and scaling down the sample amount to sub-microgram levels, making the PISA method suitable for NanoProteomics. OPTI-PISA can be implemented using only the standard equipment of a proteomics lab.

As proteins are cellular building blocks and indispensable elements of cell mechanics, most processes that occur in a cell or organism involve proteins.^1−3^ Protein higher-order structures undergo changes when proteins in a cell, bodily fluid, or isolated state interact with other molecules, including proteins,^4,5^ metabolites,^6,7^ and drug molecules.^8^ Monitoring these changes in protein structure is important to understand how cells live, metabolize food, proliferate, communicate with each other, mutate, differentiate, and die.^9−12^

In biology and medicine, it is often important to identify which protein in a whole proteome interacts with an active molecule (e.g., a drug). Thermal proteome profiling (TPP^13^), also known as CETSA-MS,^8^ is a method for identifying drug targets by measuring changes in the thermal stability of proteins in a proteome-wide manner. Protein thermal stability is probed by incubating a proteome solution at a set of temperature points varying from normal to elevated (e.g., 37 to 67 °C), centrifuging, and performing proteomic analysis of the supernatant at each temperature. At elevated temperatures, native conformations of many proteins unfold (“melt”), which makes most proteins less soluble and ultimately results in protein aggregation. TPP measures the shift in protein melting temperature (*T*_m_), which is postulated to occur when the protein binds a ligand. A typical TPP workflow involves subjecting samples to ∼10 different temperature points and using one tandem mass tag (TMT) set per sample to obtain *T*_m_ values by fitting a sigmoidal curve into the relative protein abundance at all temperature points. To establish the ligand-induced shift in *T*_m_, this procedure needs to be performed separately for drug-treated and control samples,^14^ and in order to assess statistical significance of the results, this has to be done in a number of replicates. TPP has been used quite extensively,^15−18^ but it is plagued by a limited throughput, large sample requirements, and high labor intensity. Another disadvantage of TPP is that many melting curves fit poorly to the sigmoidal shape, which prevents precise *T*_m_ measurements.^14,19^ The isothermal shift assay (iTSA) enhanced throughput over TPP by incubating samples at a single temperature for a fixed time duration.^20,21^ However, as protein melting temperatures vary widely, ranging from 40 to 60 °C,^22^ using a single temperature and incubation time may limit the applicability of this approach to a subset of proteins in the proteome.^15−18^

Proteome integral solubility alteration (PISA)^23^ has been developed as a high-throughput alternative to TPP.^5,24−26^ In PISA, the samples heated to different temperatures are combined and the overall change in protein solubility (which is equivalent to the area under the melting curve in TPP) is measured. PISA increases the analysis throughput by 1 to 2 orders of magnitude and significantly reduces the required sample amount compared to TPP.^27^ However, classical PISA is still not capable of analyzing low-microgram amounts of samples because dividing the sample into multiple vessels for thermal treatment results in significant sample loss on the walls of the tubes and pipettes. Moreover, PISA largely inherited manual labor intensity from TPP. Automation of PISA could greatly reduce the labor demand and increase the repeatability of the results. A more precise analysis would require fewer replicates to obtain the desired statistical power, which would result in a significant reduction of the analysis time and sample consumed.

Automation of PISA requires solving the problem of heating different sample portions to different temperatures and then combining these portions in a single volume. Initially, we solved this problem by injecting the sample of lysate with or without the drug into a straight capillary (“one pot”) attached to a metal plate. The plate was heated by two heaters at both ends, such that a temperature gradient existed across the plate and thus across the capillary. The injected sample stayed in a differentially heated capillary for a few minutes and then was eluted to a vial and centrifuged. Different parts of the same sample were thus exposed to different temperatures without the need to keep them in separate vessels. This approach also obviates the need for splitting the sample into different tubes for incubation at different temperatures. The approach was tested and found to be workable, showing the potential to be automated. However, maintaining a precise temperature gradient turned out to be a challenge during long runs. In a modern laboratory, the ambient air temperature is controlled by a computer that can be programmed to reduce it at night and over the weekends. As the temperature gradient on the heated PISA plate depends upon the ambient conditions, changes in air temperature may affect that gradient, resulting in experiment-to-experiment variations.

Thus, we chose an alternative approach, using the fact that protein solubility can be modulated not only by heating the sample to different temperatures for the same time duration, as in TPP and PISA, but also by heating different sample volumes to the same temperature but for different time intervals. This is somewhat similar to egg boiling, in which different boiling times result in different softness of the eggs. In that approach termed one-pot time-induced (OPTI)-PISA, the metal plate is uniformly heated to an intermediate temperature (which is much easier to maintain constant than a temperature gradient) at which different parts of the sample in a capillary are incubated for different time intervals. The latter is achieved by a fast injection of the sample into the heated capillary and a slower elution from it (Figure 1). Automated injection and elution ensure repeatable experimental conditions consistently applied to different samples, which reduce the statistical uncertainty of the results compared to manual sample handling. Also, performing incubation in a single vessel enables a dramatic reduction in sample volume.

**Figure 1:**
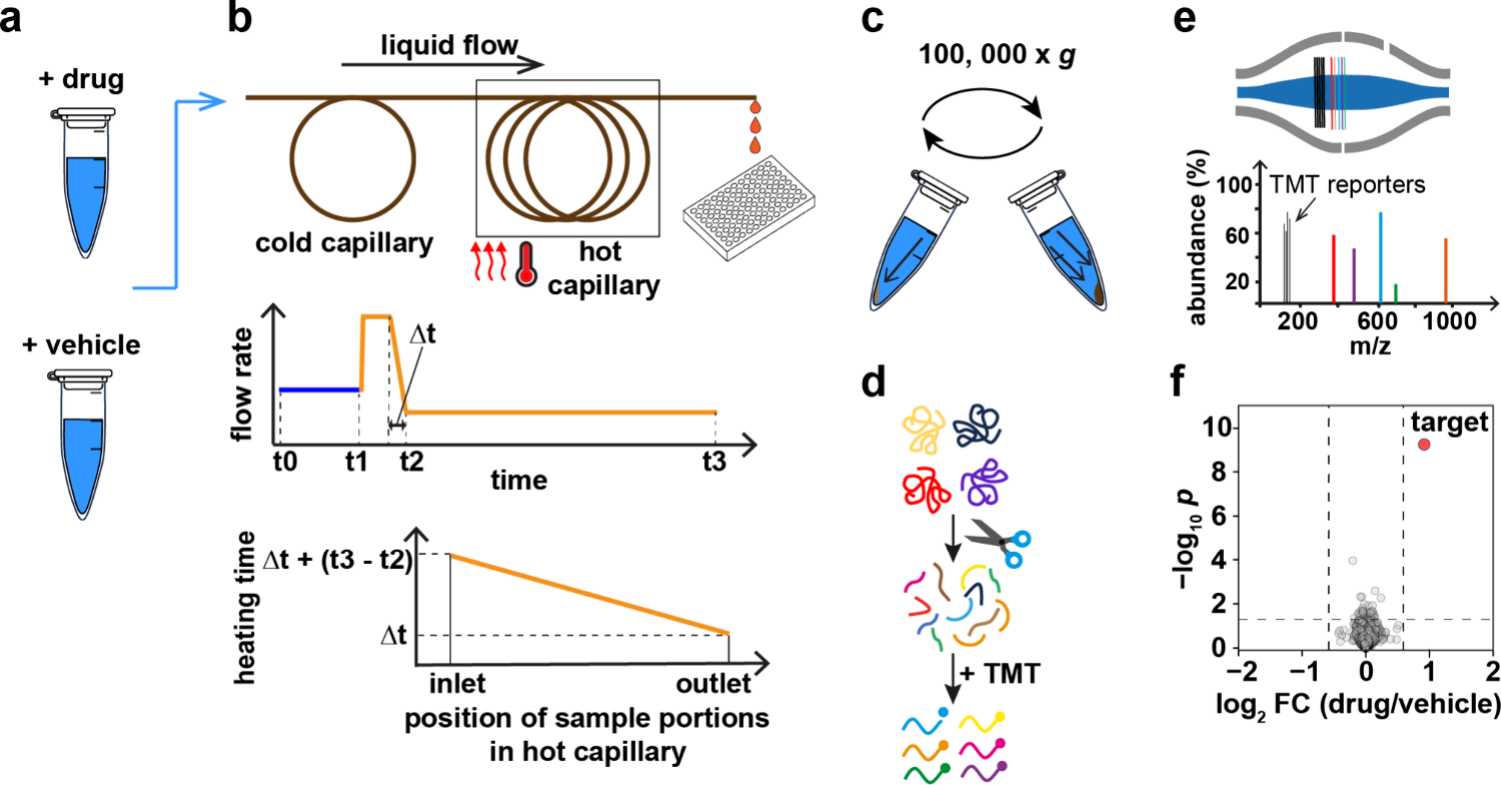
Schematic diagram of the OPTI-PISA workflow. (a) The cell lysate is treated with drug or vehicle. (b, upper) The treated sample is first injected into the cold sample loop and then transferred to a heated capillary kept at a constant temperature and, after a short time interval, eluted at a slow flow rate. (b, middle) The flow rate through the capillary. (b, lower) Exposure time of sample molecules located in different capillary parts to elevated temperature. (c) Ultracentrifugation and removal of aggregated proteins. (d) Proteomic sample preparation. (e) LC-MS/MS analysis. (f) Data processing and analysis.

In the proof of principle of the OPTI-PISA assay, we employed an automated liquid handling system equipped with a pump, autosampler, and fractionator (Biomotif) (Figure S1). A549 cell lysate (17.5 μg/sample) with and without addition of 10 μM methotrexate (MTX) was used as a model system. The results were compared between the manually controlled injection/elution and fully automatic sample processing. The plate was heated to 50 °C; the injection rate was 50 μL/min, while the elution rate was 100 times slower, 0.5 μL/min. All samples were processed in five replicates multiplexed into a single TMT-10 labeling set. In both manual and automated processing, the subsequent LC-MS/MS analysis correctly identified DHFR as a sole target among 3722 and 3291 proteins, respectively, quantified with ≥2 unique peptides (Figure 2a,b, Tables S1 and S2). As expected, OPTI-PISA automation improved the *p* value from 7 × 10^–6^ for the manual setup to 5.8 × 10^–10^ in automated sample processing. In terms of statistical significance, this improvement was equivalent to roughly doubling the number of replicates in the manual analysis.

**Figure 2:**
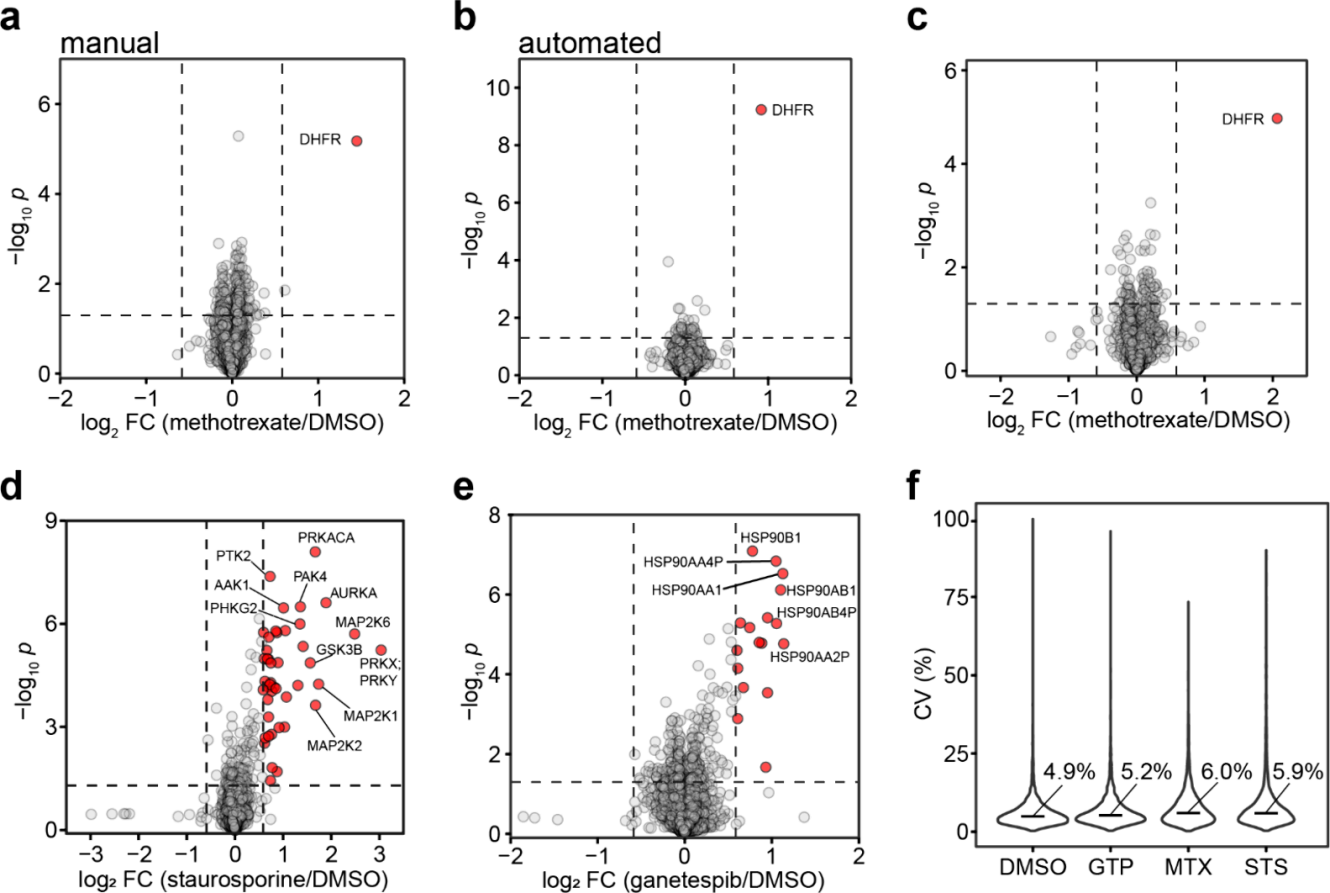
OPTI-PISA results for A549 cell lysate incubated with 10 μM drugs, versus vehicle incubation. (a–c) Lysate incubated for 1 h with drugs. (a) MTX, 17.5 μg of lysate per sample, manual injection/elution. (b–f) Automated OPTI-PISA. (b) MTX, 17.5 μg of lysate per sample. (c) MTX, 900 ng of lysate per sample. (d) Staurosporine, 150 μg of lysate per sample, incubated for 30 min. (e) Ganetespib, 150 μg of lysate per sample, incubated for 30 min. (f) CVs of protein abundances. Median values are shown.

To test the suitability of OPTI-PISA for NanoProteomics, the protein amount was reduced to 900 ng per replicate. DHFR was still identified as the sole MTX target (Figure 2c, Table S3) among the 1443 proteins quantified with ≥2 unique peptides.

To generalize the OPTI-PISA approach, we tested its performance on a standard HPLC setup equipped with an autosampler and fraction collector (Figure S2). The latter were used for liquid handling, while an external heater was used for heating of the sample inside the capillary attached to the heater. After thermal treatment, each sample was frozen in liquid nitrogen before processing. As a test system, A549 lysate was treated with 3 drugs at a 10 μM concentration and vehicle as a control, with 4 replicates of each treatment multiplexed into a single TMT-16 set. As drugs, we used staurosporine (a broad spectrum protein kinase inhibitor^28^), ganetespib (a HSP90 inhibitor^29,30^), and MTX (Figure S3a). In total, 5408 proteins were quantified with ≥2 unique peptides (Table S4). For staurosporine, 38 kinases showed a significant increase in solubility (Figure S3b). Of these, 28 (74%) were among the 268 known targets of staurosporine,^31^ while random overlap would give on average only 2 common proteins. For ganetespib, HSP90 had the most significant increase in solubility (Figure S3c), in agreement with the literature.^29^ Importantly, all median coefficients of variability (CVs) of the protein abundances across the replicates were lower than 8% (Figure S3d). This result highlighted excellent reproducibility of this OPTI-PISA setup, even though pipetting of the cell lysate into individual sample wells and adding drugs/vehicle to each sample were still manually performed.

To make OPTI-PISA completely automated, we employed the HPLC column oven for thermal treatment and programmed the autosampler to automatically pipet cell lysate and drugs/vehicle from their storage vial, while the fraction collector was instructed to store the thermally treated samples at 4 °C before further processing. The performance of the completely automated OPTI-PISA setup was done using the same test system as above. In total, 6184 proteins were quantified with ≥2 unique peptides (Table S5). The positive control, DHFR protein, was identified as the sole target of MTX (Figure S4a). The staurosporine results revealed 44 kinases with a significant increase in solubility (Figure 2d), 31 (70%) of which were among the 268 known targets of staurosporine (random overlap would produce only 2 common proteins). In ranking the quantified proteins by their log2-scaled fold change multiplied by −log10-scaled *p* value, the top 40 proteins were all kinases (Figure S4b). In comparison with the previously published TPP and PISA results for staurosporine, OPTI-PISA identified in total 42 proteins, all of them kinases, which was 6 times as many kinases identified as in TPP (7 kinases) and 2.5 times as in PISA (17 kinases), for the same *q* value of <0.05^32^ (*q* values were derived from the *p* values^33^). Notably, 71% of TPP-identified kinases and 88% of PISA-identified kinases were found in the OPTI-PISA results (Figure S5). For ganetespib, HSP90 exhibited the most significant increase in solubility (Figure 2e), while the top 6 proteins in the above ranking were all proteoforms of HSP90 (Figure S4c). The median CVs of all protein abundances across the 4 replicates were lower than 7% (Figure 2f), highlighting the superior reproducibility of completely automated OPTI-PISA.

In conclusion, the OPTI-PISA approach afforded easy and complete automation of sample thermal treatment in PISA experiments, offering higher sensitivity and repeatability with lower sample and labor requirements.

## Data Availability

The raw mass spectrometric data used in this study are available at PRIDE ProteomeXchange with the identifier PXD050241.

## Supporting Information Available

Figure S1: An automated liquid handling system for proof-of-principle OPTI-PISA experiments; Figure S2: A standard HPLC system for OPTI-PISA experiments; Figure S3: Automated OPTI-PISA results for A549 cell lysate and 10 μM drugs; Figure S4: Results from fully automated OPTI-PISA for A549 cell lysate and 10 μM drugs; Figure S5: Comparison of identified kinases between OPTI-PISA, TPP, and PISA for the drug staurosporine; Detailed description of OPTI-PISA instrumentation and experimental procedures and methods including cell culture, sample processing, proteomic sample preparation, parameters for LC–MS/MS, and data analysis (PDF)
Table S1: Manual OPTI-PISA for MTX in A549 cell lysate (XLSX)
Table S2: Automated OPTI-PISA for MTX in A549 cell lysate (XLSX)
Table S3: Automated OPTI-PISA for MTX in A549 cell lysate with 900 ng of lysate per sample (XLSX)
Table S4: Automated OPTI-PISA for multitarget drugs in A549 cell lysate (XLSX)
Table S5: Completely automated OPTI-PISA for multitarget drugs in A549 cell lysate (XLSX)
