# Supplementary material for: One-Pot Time-Induced Proteome Integral Solubility Alteration Assay for Automated and Sensitive Drug–Target Identification
Source: Anal Chem. 2024 Nov 20;96(48):18917–21. doi: 10.1021/acs.analchem.4c05127 (PMC11618734; doi:10.1021/acs.analchem.4c05127)
Supplement: Supplementary file 1 — ac4c05127_si_001.pdf [file ac4c05127_si_001.pdf]

One-Pot Time-Induced Proteome Integral Solubility Alteration Assay for Automated and Sensitive Drug–Target Identification

Supporting Information

Zhaowei Meng[1,2,3], Amir Ata Saei [4], Hezheng Lyu[1,5], Massimiliano Gaetani[1,2,3], Roman A. Zubarev[1,2,3,6*]

[1] Division of Chemistry I, Department of Medical Biochemistry and Biophysics, Karolinska Institutet, 17177 Stockholm, Sweden

[2] Chemical Proteomics Unit, Science for Life Laboratory (SciLifeLab), 17165 Stockholm, Sweden

[3] Chemical Proteomics, Swedish National Infrastructure for Biological Mass Spectrometry (BioMS), 17177 Stockholm, Sweden

[4] Department of Microbiology, Tumor and Cell Biology, Karolinska Institutet, 17177 Stockholm, Sweden

[5] Biomotif AB, 18212 Danderyd, Sweden

[6] Department of Pharmaceutical and Toxicological Chemistry, Medical Institute, Peoples' Friendship University of Russia named after Patrice Lumumba (RUDN University), 6 Miklukho-Maklaya St., Moscow 117198, Russian Federation

* Corresponding author. Email: Roman.Zubarev@ki.se

Table of Contents

Supplementary Figures S1-S5 (page S2-S6)

Methods (page S7-S12)

OPTI-PISA instrumentation and experimental procedures (page S7-S10)

LC-MS/MS (page S11-S12)

Data Processing (page S12)

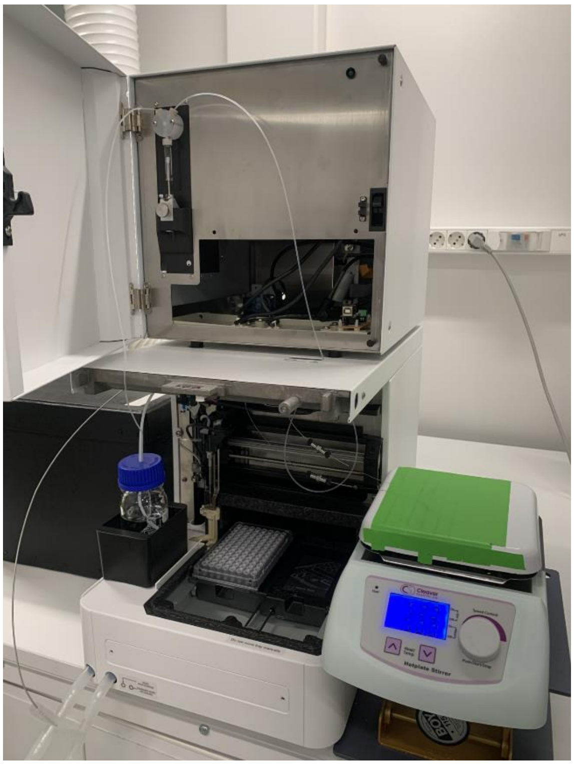

Figure S1. An automated liquid handling system for proof-of-principle OPTI-PISA experiments.

This system is equipped with a pump, autosampler and fractionator (Biomotif, Danderyd, Sweden).

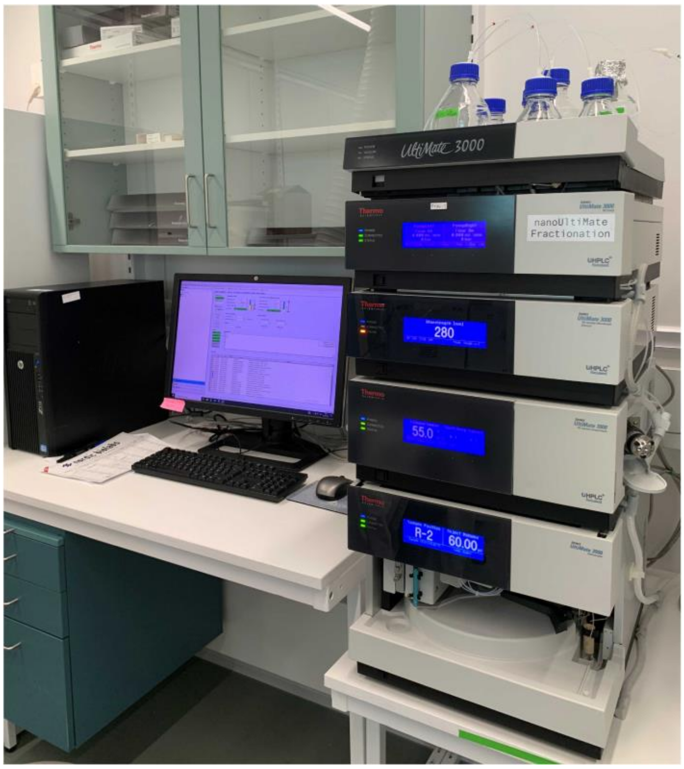

Figure S2. A standard HPLC system for OPTI-PISA experiments. This system is a widely used Thermo Ultimate 3000 HPLC equipped with an autosampler combined with fraction collector.

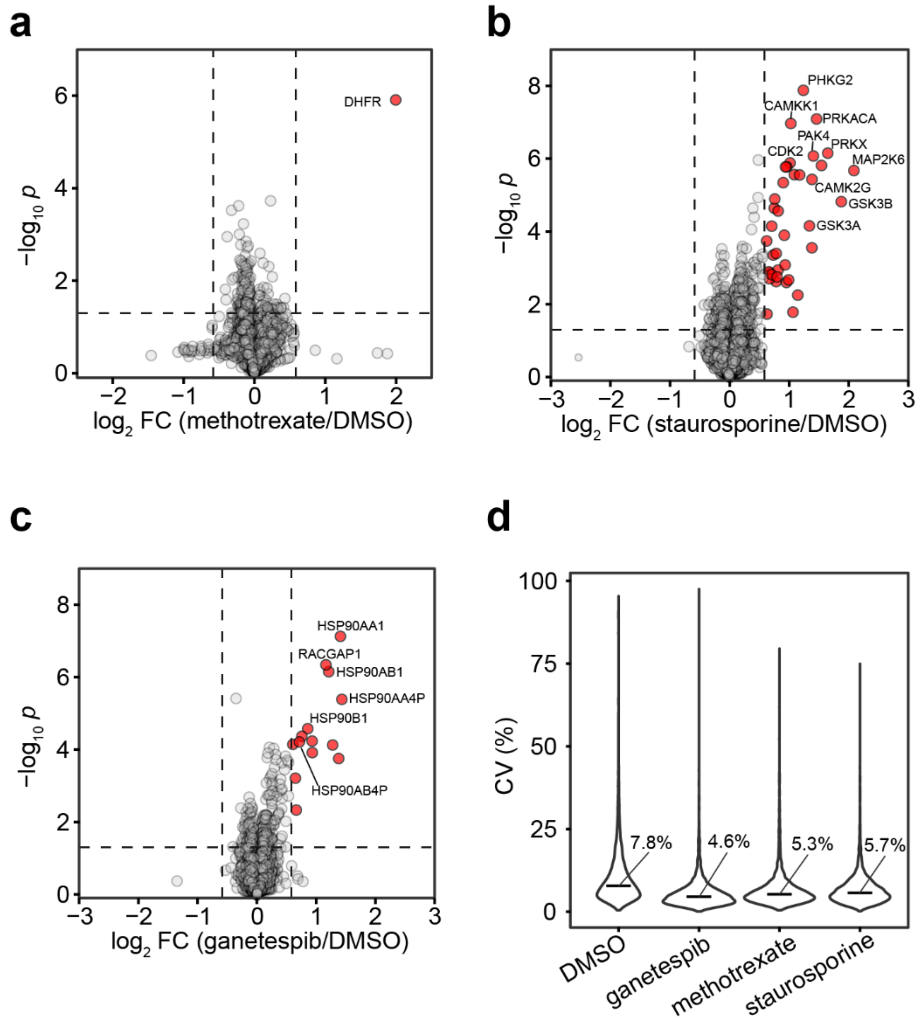

Figure S3. Automated OPTI-PISA results for A549 cell lysate and 10 µM drugs. (**a**) MTX. (**b**) Staurosporine. (**c**) Ganetespib. (**d**) CVs of protein abundances. (**a-c**) 70 µg/sample.

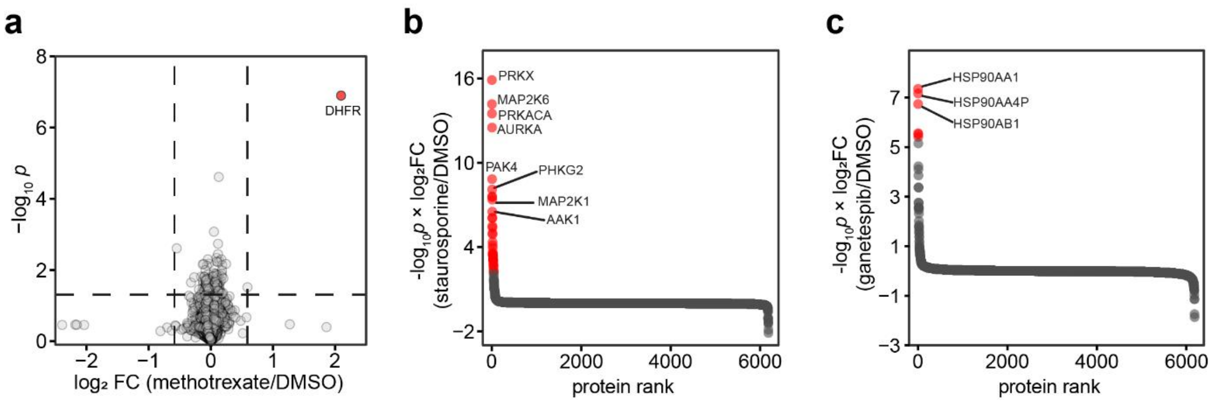

Figure S4. Results from fully automated OPTI-PISA for A549 cell lysate and 10 µM drugs. (**a**) MTX as a positive control. (**b**) Ranks of quantified proteins sorted by their log2-scaled fold change multiplied by −log10-scaled p value for staurosporine and (**c**) ganetespib.

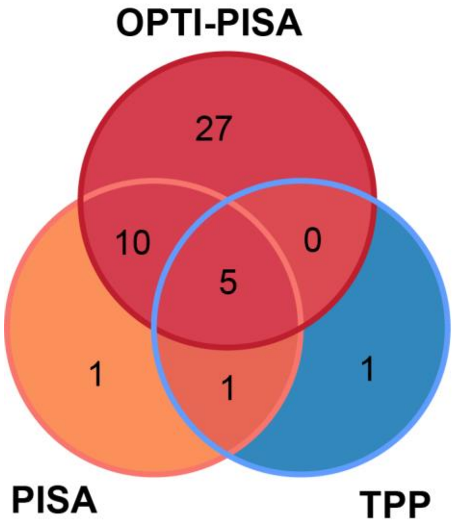

Figure S5. Comparison of identified kinases between OPTI-PISA, TPP and PISA for the drug staurosporine.

METHODS

Cell Culture. Human lung carcinoma A549 cells (ATCC) were grown at 37°C in 5% CO_2 using Dulbecco's Modified Eagle Medium (Lonza) supplemented with 10% FBS superior (Biochrom) and 100 units/mL penicillin/streptomycin (Gibco). Low-number passages (<10) were used for the experiments. Cellular lysates were obtained in PBS by five cycles of freezing cells in liquid N_2 and then thawing at 25 °C, then cleared by centrifugation at 14,000 rpm for 10 min at 4 °C. The protein concentration in the lysate was measured using Pierce BCA kit (Thermo Fisher).

Manual OPTI-PISA Configuration for MTX experiment. The device consists of a syringe pump (Chemyx), 100 µL syringes (Hamilton), connected by a union to fused silica capillary (75 µm × 113 cm, 5 µL volume) attached to a metal plate kept at a constant temperature (50 °C) by a programmable heater. The sample filled the capillary at a flow rate of 100 µL/min for 10 s, then the syringe pump stopped the flow for 10 s. Subsequently, the syringe pump eluted the sample at a flow rate of 0.5 µL/min for 10 min, and the elution was collected into a 0.5 mL-LoBind tube (Eppendorf) containing 95 µL phosphate buffered saline (PBS). The syringe and capillary were washed with PBS before running the next sample.

Automated OPTI-PISA Configuration for MTX experiments. In the manual OPTI-PISA instrument, the syringe pump was replaced by a programmable liquid sample handler comprising a pump and an autosampler with a fractionation collection option. 15 µL of sample was drawn up from a 96-well plate into a 10 µL sample loop. The pump pushed PBS at 20 µL/min to wash capillary for 2 min, and fast transferred the sample from the sample loop into the heated capillary at a flow rate of 50 µL/min for 8 s, then stopped for 10 s for stabilizing the flow. Then the pump eluted the sample from the hot capillary into the 96-well plate at a flow rate of 0.5 µL/min for 5 min, and then switched to another well for 10 min. The aim is to collect two fractions per sample, one (≈5 µL) subjected to intended thermal treatment that will be used for analysis, and another one wasted as an overhead. For testing the suitability of OPTI-PISA for NanoProteomics, sample amount was reduced to 900 ng of

lysate per replicate, capillary temperature was increased from 50 °C to 55 °C while elution flow rate was increased from 0.5 µL/min to 1.0 µL/min.

Automated OPTI-PISA Configuration for Multiple Drug experiment. Ultimate 3000 HPLC system with fractionation option (Thermo Fisher) was employed. As an external device, a fused silica capillary (250 µm × 102 cm, 50 µL volume) was placed on a metal plate and heated to 55 °C by a programmable thermal heater. The autosampler picked 70 µL of sample into a 125 µL sample loop, and the pump fast transferred sample from sample loop to the heating capillary at a flow rate of 200 µL/min for 0.25 min, then reduced the flow rate to 10 µL/min to elute the sample from the hot capillary into a 96-well plate.

Completely Automated OPTI-PISA Configuration for Multiple Drug experiment. Ultimate 3000 HPLC system with fractionation option (Thermo Fisher) was employed. A fused silica capillary (380 µm × 89 cm, 100 µL volume) was placed in HPLC column oven heated to 55 °C. The autosampler picked 100 µL of sample into a 125 µL sample loop, and the pump fast transferred sample from sample loop to the heating capillary at a flow rate of 200 µL/min for 0.63 min, then reduced the flow rate to 20 µL/min to elute the sample from the hot capillary into a 96-well plate.

OPTI-PISA Experiments with MTX. A549 cellular lysate was treated with MTX at 10 µM final concentration or an equal amount of DMSO (control) with 5 replicates in PBS for 60 min at 25°C. Samples were respectively subjected to thermal treatment on the manual OPTI-PISA configuration or automated OPTI-PISA instrument as described above. Collected sample elution was then left at room temperature for 4 min before being snap frozen in liquid N_2. The samples were then transferred into polycarbonate thick-wall tubes and centrifuged at 100,000 g at 4 °C for 30 min. The supernatant was collected. Lys-C (Wako) was added at a 1:40 w/w ratio and samples were incubated at 30 °C overnight. Sequencing grade modified trypsin (Promega) was then added at a 1:20 w/w ratio, and the samples were digested at 37 °C for 4 h. TMT 10plex reagents dissolved in acetonitrile (ACN) were added 4× by weight to each sample with a final ACN concentration of 30%, followed by incubation for 2 h at

25 °C. The reaction was quenched by adding 0.5% hydroxylamine. Samples were combined, acidified by TFA to pH 1-3. Sample desalting and fractionation was performed by high-pH reverse-phase fractionation Kit (Thermo Fisher). A total of 12 fractions were collected and concatenated into 10 fractions. Sub-microgram samples were cleaned by StageTips without fractionation after TMT-10 labelling. Samples were dried in a DNA 120 SpeedVac Concentrator (Thermo Fisher) and stored at −80 °C until LC-MS/MS analysis.

OPTI-PISA Experiment with Multi-target Drugs. 3 drugs (methotrexate, staurosporine, ganetespib) at a final concentration of 10 μM and DMSO (control) were used for treating the A549 lysate in 4 replicates. The A549 lysate (1.5 µg/µL) was distributed into 16 equal aliquots in sample vials (80 µL/vial) and these vials were stored at 4 °C. 1 µL of 800 µM drug or same concentration of DMSO was added to each sample vial containing 80 µL of A549 lysate, pipetting 10 times without making bubbles. After incubating at 25 °C for 15 min, the sample was deposited in the autosampler of Ultimate 3000 HPLC system for OPTI-PISA experiment as described above. The eluted sample was left at room temperature for 4 min before being snap frozen in liquid N_2. Samples were then transferred into polycarbonate thick-wall tubes and centrifuged at 100,000 × g for 30 min at 4 °C. The supernatant was collected, and its protein concentration was measured using Pierce BCA kit. Dithiothreitol (DTT) was added to a final concentration of 8 mM and samples were incubated for 45 min at 55 °C. Subsequently, iodoacetamide (IAA) was added to a final concentration of 25 mM and samples were incubated at 25 °C for 30 min in the darkness. Proteins were precipitated using cold acetone at a sample: acetone ratio of 1:6 (v/v) at −20 °C overnight. The precipitated protein was collected by centrifugation (10,000 × g, 10 min), and the supernatant was removed. The protein pellet was air-dried for 3 min, then dissolved in 10 µL of 20 mM EPPS buffer at pH 8.2 containing 8 M urea for 10 min. 20 mM EPPS buffer (pH 8.2) was added to dilute urea to 4 M. Then 1 µL of LysC was added at a 1:75 w/w ratio, the samples were incubated at 30 °C for 6 h. The samples were diluted with 20 mM EPPS to a final urea concentration of 1 M, and trypsin was added at a 1:50 w/w ratio, followed by incubation at 37 °C overnight. TMTpro 16plex reagents dissolved in acetonitrile (ACN) were added 5× by weight to each sample with a final ACN concentration of 30%, followed by incubation for 2 h at 25 °C. The reaction

was quenched by adding 0.5% hydroxylamine. Samples were combined, acidified by TFA to pH 1-3, cleaned using Sep-Pak cartridges (Waters), and dried using DNA 120 SpeedVac Concentrator (Thermo Fisher). Peptide separation for deeper proteome analysis was carried out using an Ultimate 3000 HPLC system (Thermo Fisher) equipped with a Xbridge Peptide BEH C18 column (25 cm × 2.1 mm, particle size 3.5 μm, pore size 300 Å; Waters) operating at a flow rate of 200 μL/min. Fractionation was achieved through a binary solvent system comprising 20 mM NH_4OH in H_2O (solvent A) and 20 mM NH_4OH in acetonitrile (solvent B). The elution profile was programmed as follows (data for % of solvent B): 2% to 23% in 42 min, 23 to 52% in 4 min, 52 to 63% in 2 min, and a subsequent isocratic hold at 63% for 5 min. The elution process was monitored by continuous of UV absorbance at 214 nm. A total of 96 fractions, each containing a 100 μL aliquot, were collected. Fractions were then concatenated into 24 samples in a sequential order (e.g. 1, 25, 49, 73).

Completely Automated OPTI-PISA Experiment with Multi-target Drugs. An Ultimate 3000 HPLC system was employed to perform all the processes in an automatic manner. 3 drugs (methotrexate, staurosporine, ganetespib) at a final concentration of 10 μM and DMSO (control) were used for treating the A549 lysate in 4 replicates. The A549 lysate (1.5 μg/μL) was automatically distributed into 16 equal aliquots in the wells of a 96-well plate (108 μL/well) and these vials were stored at 4 °C. 12 μL of 100 μM drug or same concentration of DMSO was automatically added to each sample well containing 108 μL of A549 lysate, shaking by the autosampler tray without making bubbles. After incubating at 25 °C for 30 min, the sample was processed for completely automated OPTI-PISA experiment as described above. Samples were then transferred into polycarbonate thick-wall tubes and centrifuged at 100,000 g for 30 min at 4 °C. The proteomics sample preparation was the same as described in **OPTI-PISA Experiment with Multi-target Drugs**. After high-pH fractionation, fractions were then concatenated into 48 samples in a sequential order (e.g. 1 and 49, 2 and 50).

LC-MS/MS Analysis

Each sample was analysed by LC-MS/MS using a Q Exactive HF or Orbitrap Exploris 480 mass spectrometers equipped with an EASY Spray Ion Source and connected to an Ultimate 3000 RSLC nano HPLC system (all - Thermo Fisher). Injected sample fractions were preconcentrated and further desalted on a trap column (Acclaim PepMap 100 C18, 75 µm × 2 cm; particle size, 3 µm; pore size, 100 Å), and then separated on an EASY Spray analytical column (Acclaim PepMap RSLC, 50 cm × 75 µm; particle size, 2 µm; pore size, 100 Å) at 55°C and a flow rate of 300 nL/min. Peptides were separated using a binary solvent system consisting of 0.1% (v/v) formic acid (FA), 2% (v/v) acetonitrile (ACN) (solvent A) and 98% ACN (v/v), 0.1% (v/v) FA (solvent B).

For the fractionated samples, the gradient consisted of 3−28% B in 115 min, 28−40% B in 5 min, 40−95% B in 5 min, 95% B for 8 min, and 95-3% B in 2 min. For the unfractionated sub-microgram sample, the gradient consisted of 3−28% B in 230 min, 28−40% B in 10 min, 40−95% B in 5 min, 95% B for 10 min, and 95-3% B in 2 min.

For the fractionated samples from OPTI-PISA experiment with single-target drug (MTX), mass spectra were acquired using Q Exactive HF. A full MS (MS1) spectrum was first acquired in the Orbitrap analyzer with mass-to-charge ratio (m/z) range from 375 to 1500, nominal resolution 120,000, automated gain control (AGC) target 3×10^6, and maximum injection time of 100 ms. Following this, the 17 most abundant peptide ions were selected for higher-energy collision dissociation (HCD) with normalized collision energy 33 for subsequent MS/MS (MS2) analysis with a minimum intensity threshold of 2×10^4 and a 30 s dynamic exclusion time. MS2 spectra were acquired in the Orbitrap analyzer with the following settings: quadrupole isolation window 1.2 Th, AGC target 2×10^5, maximum injection time 120 ms, nominal resolution 45,000, and fixed first m/z 110.

For the fractionated samples obtained from OPTI-PISA assay for 3 drugs, mass spectra were acquired using Orbitrap Exploris 480. A full MS (MS1) spectrum was first acquired with m/z range from 375 to 1500, nominal resolution 120,000, AGC target 3×10^6, and maximum injection time 50 ms. The most abundant peptide ions were selected for HCD with normalized collision energy 35 for

subsequent MS/MS (MS2) analysis with a minimum intensity threshold of 5×10^4 and a 30 s dynamic exclusion time. MS2 spectra were acquired at nominal resolution 45,000, with AGC target 2.5×10^5 and maximum injection time 120 ms. The fixed first m/z was 110 and the isolation window was 0.7 Th. The number of MS2 spectra acquired per each MS1 spectrum was determined by setting the maximum cycle time for MS1 and MS2 spectra to 3 s (using the top speed mode).

For the unfractionated sub-microgram sample, Orbitrap Exploris 480 was used for mass spectra acquisition. All the parameters were the same as used for the fractionated samples obtained from OPTI-PISA with 3 drugs, except for normalized collision energy set to 33 and dynamic exclusion time 60 s.

Data Processing

The raw LC-MS/MS data were analysed by MaxQuant, version 2.2.0.0. The Andromeda search engine was employed to perform MS/MS data matching against the UniProt Human proteome database (version UP000005640_9606, 20607 human protein sequences). Enzyme specificity was set to trypsin, with maximum two missed cleavages permitted. Cysteine carbamidomethylation was set as a fixed modification, while methionine oxidation, N-terminal acetylation, asparagine or glutamine deamidation were used as variable modifications. 1% false discovery rate was used as a filter at both protein and peptide levels. Default settings were employed for all other parameters. Peptide quantification was executed using the abundances of reporter ions of TMT 10 plex or TMTpro 16plex according to the experiments. The obtained protein abundances were normalized on the total ion abundance of TMT reporters for a given protein, then the average value of protein abundance across replicates was calculated. Volcano plots were generated by calculating the protein abundance fold change (FC) between drug and DMSO treated samples and performing Student's t-tests for the difference between the two treatment groups.
